# 1-Furoyl-3-methyl-3-phenyl­thio­urea

**DOI:** 10.1107/S1600536807068687

**Published:** 2008-01-25

**Authors:** Hiram Pérez, Yvonne Mascarenhas, Osvaldo Estévez-Hernández, Sauli Santos Jr, Julio Duque

**Affiliations:** a Departamento de Química Inorgánica, Facultad de Química, Universidad de la Habana, Habana 10400, Cuba; b Instituto de Física de São Carlos, Universidade de São Paulo, São Carlos, Brazil; c Instituto de Ciencia y Tecnología de Materiales, Universidad de la Habana, Habana 10400, Cuba; d Laboratório de Física, Universidade Federal do Tocantins, CEP 77020-120, Palmas, Tocantins, Brazil

## Abstract

The title compound, C_13_H_12_N_2_O_2_S, crystallizes with two independent mol­ecules in the asymmetric unit. The two mol­ecules differ in the conformation of the thio­carbonyl and carbonyl groups, and show the typical geometric parameters of substituted thio­urea derivatives. The crystal structure is mainly stabilized by inter­molecular N—H⋯O hydrogen bonding.

## Related literature

For general background, see: Estévez-Hernández *et al.* (2007 ▶); Otazo *et al.* (2001 ▶). For related structures, see: Koch *et al.* (1995 ▶); Morales *et al.* (1997 ▶). For synthesis, see: Otazo *et al.* (2001 ▶).
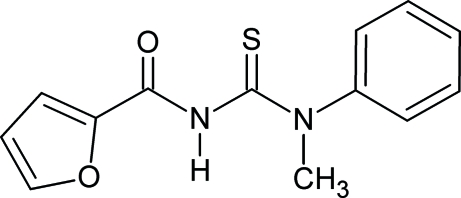


## Experimental

### 

#### Crystal data

C_13_H_12_N_2_O_2_S
*M* *_r_* = 260.31
Monoclinic, 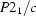

*a* = 10.242 (1) Å
*b* = 13.525 (1) Å
*c* = 18.432 (2) Å
β = 96.115 (4)°
*V* = 2538.7 (4) Å^3^
*Z* = 8
Mo *K*α radiation
μ = 0.25 mm^−1^
*T* = 150 (2) K
0.12 × 0.08 × 0.06 mm

#### Data collection

Nonius KappaCCD diffractometer
Absorption correction: none
23939 measured reflections
4440 independent reflections
2828 reflections with *I* > 2σ(*I*)
*R*_int_ = 0.093

#### Refinement

*R*[*F* ^2^ > 2σ(*F* ^2^)] = 0.046
*wR*(*F* ^2^) = 0.117
*S* = 1.01
4440 reflections
327 parameters
H-atom parameters constrained
Δρ_max_ = 0.26 e Å^−3^
Δρ_min_ = −0.39 e Å^−3^

### 

Data collection: *COLLECT* (Nonius, 2000 ▶); cell refinement: *HKL* *SCALEPACK* (Otwinowski & Minor, 1997 ▶); data reduction: *HKL* *DENZO* (Otwinowski & Minor, 1997 ▶) and *SCALEPACK*; program(s) used to solve structure: *SHELXS97* (Sheldrick, 2008 ▶); program(s) used to refine structure: *SHELXL97* (Sheldrick, 2008 ▶); molecular graphics: *ORTEP-3 for Windows* (Farrugia, 1997 ▶); software used to prepare material for publication: *WinGX* (Farrugia, 1999 ▶).

## Supplementary Material

Crystal structure: contains datablocks global, I. DOI: 10.1107/S1600536807068687/xu2395sup1.cif

Structure factors: contains datablocks I. DOI: 10.1107/S1600536807068687/xu2395Isup2.hkl

Additional supplementary materials:  crystallographic information; 3D view; checkCIF report

## Figures and Tables

**Table 1:** Hydrogen-bond geometry (Å, °)

*D*—H⋯*A*	*D*—H	H⋯*A*	*D*⋯*A*	*D*—H⋯*A*
N2—H2⋯O3^i^	0.88	2.54	3.331 (3)	149
N4—H4⋯O1^ii^	0.88	2.17	3.010 (2)	159

Symmetry codes: (i) 
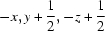
; (ii) 
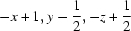
.

**Fig. 1.**
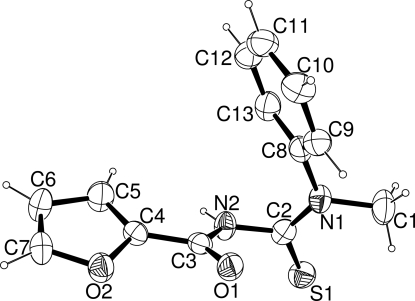
The molecular structure of title compound. Displacement ellipsoids are drawn at the 50% probability level.

**Fig. 2.**
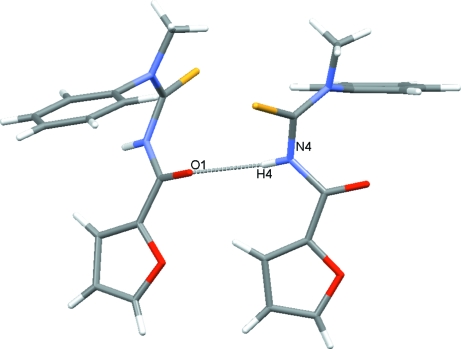
Intermolecular interaction observed between asymmetric units. Hydrogen-bonding is indicated by a dashed line.

## supplementary crystallographic information

### Comment

Substituted *N*-acylthioureas have been a subject of investigations, due to their ability to form stable metal complexes and as ionophores in potenciometric and amperometric sensors for Cd(II), Hg(II) and Pb(II) (Otazo *et al.*, 2001; Estévez-Hernández *et al.*, 2007). The title compound, (I) (Fig. 1), is another example of our newly synthesized furoylthiourea derivatives, which shows outstanding complexation properties.

The main bond lengths and angles are given in Table 1, and are within the ranges obtained for similar compounds (Koch *et al.*, 1995; Morales *et al.*, 1997). The C—S and C3—O1, C16—O3 bonds show typical double-bond character. However, the C—N bond lengths, C2—N1, C2—N2, C3—N2, and the corresponding lengths for the other molecule are shorter than the normal C—N single-bond length of about 1.48 Å. These results can be explained by the existence of resonance in this part of the molecule. The crystal structure is stabilized by intermolecular N2—H2···O3 and N4—H4···O1 hydrogen-bonds (Table 2) between asymmetric units (Fig. 2).

The dihedral angles of two independent molecules between the furan and benzene ring planes are 67.8 (1)° and 82.8 (1)°, respectively. In addition, the conformation with respect to the thiocarbonyl and carbonyl moieties is twisted, as reflected by the torsion angles O1—C3—N2—C2 and C3—N2—C2—N1 of 0.3 (4) and -66.0 (3)° for one molecule, and O3—C16—N4—C15 and C16—N4—C15—N3 of -21.2 (4) and 61.9 (3)° for the other one.

### Experimental

The title compound, (I), was synthesized according to a procedure described by Otazo *et al.* (2001), by converting furoyl chloride into furoyl isothiocyanate and then condensing with the appropriate amine. The resulting solid product was crystallized from a dichlorometane-methanol (1:1) mixture yielding X-ray quality single crystals (m.p. 374.5 K). Elemental analysis for C_13_H_12_N_2_O_2_S found: C 55.7, H 7.5, N 9.6, S 22.1%; calculated: C 56.34, H 7.43, N 9.4, S 21.5%.

### Refinement

H atoms were placed in calculated positions with N—H = 0.88 Å and C—H = 0.95 Å (aromatic) or 0.98 Å (methyl), and refined in riding model, *U*_iso_(H) = 1.5*U*_eq_(C) for methyl and 1.2*U*_eq_(C,*N*) for others.

### Crystal data

**Table:**

C_13_H_12_N_2_O_2_S	*F*_000_ = 1088
*M**_r_* = 260.31	*D*_x_ = 1.362 Mg m^−^^3^
Monoclinic, *P*2_1_/*c*	Mo *K*α radiation λ = 0.71073 Å
Hall symbol: -P 2ybc	Cell parameters from 17369 reflections
*a* = 10.242 (1) Å	θ = 2.9–26.0º
*b* = 13.525 (1) Å	µ = 0.25 mm^−^^1^
*c* = 18.432 (2) Å	*T* = 150 (2) K
β = 96.115 (4)º	Prism, colourless
*V* = 2538.7 (4) Å^3^	0.12 × 0.08 × 0.06 mm
*Z* = 8	

### Data collection

**Table:**

Nonius KappaCCD diffractometer	*R*_int_ = 0.093
CCD scans	θ_max_ = 25.0º
Absorption correction: none	θ_min_ = 3.2º
23939 measured reflections	*h* = −12→12
4440 independent reflections	*k* = −15→16
2828 reflections with *I* > 2σ(*I*)	*l* = −21→21

### Refinement

**Table:**

Refinement on *F*^2^	H-atom parameters constrained
Least-squares matrix: full	*w* = 1/[σ^2^(*F*_o_^2^) + (0.0586*P*)^2^ + 0.0231*P*] where *P* = (*F*_o_^2^ + 2*F*_c_^2^)/3
*R*[*F*^2^ > 2σ(*F*^2^)] = 0.046	(Δ/σ)_max_ = 0.004
*wR*(*F*^2^) = 0.117	Δρ_max_ = 0.26 e Å^−^^3^
*S* = 1.01	Δρ_min_ = −0.39 e Å^−^^3^
4440 reflections	Extinction correction: none
327 parameters	

### Special details

**Table:**

Geometry. All e.s.d.'s (except the e.s.d. in the dihedral angle between two l.s. planes) are estimated using the full covariance matrix. The cell e.s.d.'s are taken into account individually in the estimation of e.s.d.'s in distances, angles and torsion angles; correlations between e.s.d.'s in cell parameters are only used when they are defined by crystal symmetry. An approximate (isotropic) treatment of cell e.s.d.'s is used for estimating e.s.d.'s involving l.s. planes.

### Fractional atomic coordinates and isotropic or equivalent isotropic displacement parameters (Å^2^)

**Table:**

	*x*	*y*	*z*	*U*_iso_*/*U*_eq_	
C1	0.2294 (3)	0.8708 (2)	0.23749 (18)	0.0532 (8)	
H1A	0.2926	0.8939	0.2049	0.08*	
H1B	0.2749	0.8593	0.2863	0.08*	
H1C	0.1611	0.921	0.2403	0.08*	
C14	0.2728 (3)	0.3832 (2)	0.26750 (17)	0.0560 (9)	
H14A	0.3457	0.4205	0.2506	0.084*	
H14B	0.2145	0.4285	0.2903	0.084*	
H14C	0.2234	0.3502	0.2259	0.084*	
C2	0.1449 (2)	0.76315 (19)	0.13688 (16)	0.0351 (7)	
C15	0.3838 (2)	0.22823 (19)	0.29748 (14)	0.0339 (6)	
C3	0.1874 (2)	0.58628 (18)	0.12911 (14)	0.0302 (6)	
C16	0.3287 (2)	0.10391 (18)	0.38668 (14)	0.0309 (6)	
C4	0.1288 (2)	0.49232 (18)	0.10477 (14)	0.0298 (6)	
C17	0.3758 (2)	0.00693 (18)	0.41385 (14)	0.0302 (6)	
C5	0.0051 (2)	0.4590 (2)	0.09248 (17)	0.0452 (8)	
H5	−0.0726	0.4963	0.0957	0.054*	
C18	0.4789 (2)	−0.04906 (19)	0.40147 (15)	0.0355 (7)	
H18	0.547	−0.0316	0.3727	0.043*	
C6	0.0122 (3)	0.3574 (2)	0.07366 (17)	0.0450 (8)	
H6	−0.0592	0.3132	0.0626	0.054*	
C19	0.4675 (2)	−0.13922 (19)	0.43928 (16)	0.0386 (7)	
H19	0.5266	−0.1935	0.4413	0.046*	
C7	0.1390 (3)	0.3370 (2)	0.07479 (15)	0.0398 (7)	
H7	0.173	0.274	0.0637	0.048*	
C20	0.3574 (3)	−0.13276 (19)	0.47157 (16)	0.0396 (7)	
H20	0.3249	−0.1833	0.5006	0.047*	
C8	0.1275 (2)	0.70935 (19)	0.26202 (15)	0.0341 (6)	
C21	0.3303 (2)	0.33719 (18)	0.39653 (15)	0.0341 (6)	
C9	0.2181 (2)	0.6730 (2)	0.31625 (16)	0.0389 (7)	
H9	0.3079	0.6915	0.3179	0.047*	
C22	0.4509 (3)	0.34928 (19)	0.43744 (17)	0.0437 (7)	
H22	0.5303	0.3339	0.4175	0.052*	
C10	0.1775 (3)	0.6096 (2)	0.36825 (16)	0.0463 (8)	
H10	0.2396	0.5842	0.4056	0.056*	
C23	0.4529 (4)	0.3848 (2)	0.5091 (2)	0.0604 (10)	
H23	0.5343	0.393	0.5384	0.072*	
C11	0.0467 (3)	0.5832 (2)	0.36605 (17)	0.0445 (7)	
H11	0.0191	0.5394	0.4017	0.053*	
C24	0.3370 (4)	0.4078 (2)	0.53737 (19)	0.0632 (10)	
H24	0.3388	0.4324	0.5857	0.076*	
C12	−0.0436 (3)	0.6203 (2)	0.31213 (18)	0.0451 (8)	
H12	−0.1333	0.6017	0.3106	0.054*	
C25	0.2198 (4)	0.3950 (2)	0.4957 (2)	0.0555 (9)	
H25	0.1403	0.4111	0.5152	0.067*	
C13	−0.0042 (2)	0.6846 (2)	0.26012 (16)	0.0415 (7)	
H13	−0.0666	0.7113	0.2236	0.05*	
C26	0.2157 (3)	0.3593 (2)	0.42587 (17)	0.0428 (7)	
H26	0.1335	0.3498	0.3977	0.051*	
O1	0.29958 (15)	0.59531 (12)	0.15853 (10)	0.0392 (5)	
O3	0.21895 (15)	0.13554 (12)	0.39322 (10)	0.0390 (5)	
O2	0.21429 (15)	0.41764 (12)	0.09384 (10)	0.0385 (5)	
O4	0.29752 (15)	−0.04324 (13)	0.45720 (10)	0.0399 (5)	
N1	0.16896 (19)	0.77824 (15)	0.20905 (13)	0.0362 (5)	
N3	0.32476 (19)	0.30851 (16)	0.32120 (12)	0.0370 (6)	
N2	0.10521 (19)	0.66592 (15)	0.11680 (12)	0.0352 (6)	
H2	0.0251	0.6562	0.0957	0.042*	
N4	0.41900 (18)	0.15394 (15)	0.35015 (12)	0.0324 (5)	
H4	0.5026	0.139	0.36	0.039*	
S1	0.16001 (7)	0.84691 (5)	0.07255 (4)	0.0453 (2)	
S2	0.41662 (7)	0.21011 (6)	0.21239 (4)	0.0489 (2)	

### Atomic displacement parameters (Å^2^)

**Table:**

	*U*^11^	*U*^22^	*U*^33^	*U*^12^	*U*^13^	*U*^23^
C1	0.068 (2)	0.0363 (18)	0.054 (2)	−0.0138 (14)	−0.0010 (16)	−0.0080 (15)
C14	0.077 (2)	0.052 (2)	0.039 (2)	0.0213 (16)	0.0060 (16)	0.0122 (16)
C2	0.0294 (13)	0.0317 (16)	0.044 (2)	0.0030 (11)	0.0016 (12)	0.0027 (14)
C15	0.0277 (13)	0.0402 (17)	0.0344 (17)	0.0004 (11)	0.0063 (11)	0.0031 (14)
C3	0.0323 (15)	0.0325 (16)	0.0268 (16)	0.0011 (11)	0.0080 (11)	−0.0008 (12)
C16	0.0300 (14)	0.0341 (16)	0.0286 (17)	−0.0020 (11)	0.0037 (11)	−0.0026 (12)
C4	0.0317 (14)	0.0283 (15)	0.0295 (16)	0.0059 (11)	0.0031 (11)	0.0014 (12)
C17	0.0274 (13)	0.0321 (15)	0.0308 (17)	−0.0074 (11)	0.0014 (11)	0.0004 (12)
C5	0.0313 (15)	0.0412 (18)	0.062 (2)	0.0021 (12)	0.0024 (13)	−0.0063 (16)
C18	0.0315 (14)	0.0323 (16)	0.0444 (18)	−0.0015 (12)	0.0122 (12)	0.0007 (14)
C6	0.0445 (17)	0.0326 (17)	0.056 (2)	−0.0088 (12)	−0.0020 (14)	−0.0038 (15)
C19	0.0378 (15)	0.0346 (17)	0.0436 (19)	0.0020 (12)	0.0062 (13)	−0.0012 (14)
C7	0.0495 (18)	0.0266 (16)	0.0420 (19)	−0.0019 (13)	−0.0013 (13)	−0.0048 (13)
C20	0.0490 (17)	0.0304 (16)	0.0389 (19)	−0.0007 (12)	0.0028 (13)	0.0062 (13)
C8	0.0402 (15)	0.0288 (15)	0.0334 (17)	−0.0011 (11)	0.0045 (12)	−0.0053 (13)
C21	0.0412 (15)	0.0284 (15)	0.0334 (18)	−0.0001 (11)	0.0070 (12)	0.0053 (13)
C9	0.0358 (15)	0.0438 (17)	0.0376 (18)	−0.0029 (12)	0.0066 (13)	−0.0033 (14)
C22	0.0453 (17)	0.0353 (17)	0.049 (2)	−0.0010 (13)	−0.0010 (14)	0.0050 (15)
C10	0.0451 (17)	0.053 (2)	0.041 (2)	0.0050 (14)	0.0053 (14)	0.0009 (16)
C23	0.084 (3)	0.0342 (19)	0.056 (3)	−0.0078 (16)	−0.0251 (19)	0.0051 (17)
C11	0.0477 (17)	0.0416 (18)	0.047 (2)	0.0020 (13)	0.0177 (14)	−0.0003 (15)
C24	0.123 (3)	0.0331 (19)	0.035 (2)	0.0044 (19)	0.012 (2)	0.0034 (15)
C12	0.0379 (16)	0.0438 (18)	0.055 (2)	−0.0028 (13)	0.0122 (14)	−0.0054 (16)
C25	0.081 (2)	0.0375 (19)	0.052 (2)	0.0048 (16)	0.0279 (19)	0.0048 (17)
C13	0.0358 (15)	0.0379 (17)	0.050 (2)	0.0019 (12)	0.0020 (13)	−0.0054 (15)
C26	0.0473 (16)	0.0383 (18)	0.045 (2)	0.0007 (13)	0.0127 (14)	0.0042 (15)
O1	0.0276 (10)	0.0407 (11)	0.0491 (13)	−0.0014 (8)	0.0033 (8)	−0.0089 (9)
O3	0.0271 (10)	0.0377 (11)	0.0534 (14)	0.0017 (8)	0.0096 (8)	0.0049 (9)
O2	0.0369 (10)	0.0320 (11)	0.0463 (13)	0.0014 (8)	0.0033 (8)	−0.0040 (9)
O4	0.0359 (10)	0.0405 (12)	0.0448 (13)	0.0017 (8)	0.0111 (8)	0.0081 (9)
N1	0.0414 (12)	0.0281 (13)	0.0381 (16)	−0.0031 (9)	−0.0002 (10)	−0.0016 (11)
N3	0.0417 (12)	0.0370 (14)	0.0327 (15)	0.0092 (10)	0.0060 (10)	0.0061 (11)
N2	0.0304 (11)	0.0299 (13)	0.0431 (15)	0.0005 (9)	−0.0060 (9)	−0.0039 (11)
N4	0.0249 (11)	0.0361 (13)	0.0367 (15)	0.0017 (9)	0.0051 (9)	0.0079 (11)
S1	0.0509 (4)	0.0379 (4)	0.0480 (5)	0.0009 (3)	0.0090 (3)	0.0061 (4)
S2	0.0498 (4)	0.0634 (6)	0.0351 (5)	0.0115 (4)	0.0115 (3)	0.0031 (4)

### Geometric parameters (Å, °)

**Table:**

C1—N1	1.468 (3)	C19—H19	0.95
C1—H1A	0.98	C7—O2	1.360 (3)
C1—H1B	0.98	C7—H7	0.95
C1—H1C	0.98	C20—O4	1.370 (3)
C14—N3	1.473 (3)	C20—H20	0.95
C14—H14A	0.98	C8—C9	1.380 (4)
C14—H14B	0.98	C8—C13	1.386 (3)
C14—H14C	0.98	C8—N1	1.446 (3)
C2—N1	1.342 (3)	C21—C26	1.376 (4)
C2—N2	1.414 (3)	C21—C22	1.387 (4)
C2—S1	1.659 (3)	C21—N3	1.437 (3)
C15—N3	1.339 (3)	C9—C10	1.383 (4)
C15—N4	1.417 (3)	C9—H9	0.95
C15—S2	1.657 (3)	C22—C23	1.404 (5)
C3—O1	1.223 (3)	C22—H22	0.95
C3—N2	1.371 (3)	C10—C11	1.383 (4)
C3—C4	1.456 (3)	C10—H10	0.95
C16—O3	1.221 (3)	C23—C24	1.381 (5)
C16—N4	1.378 (3)	C23—H23	0.95
C16—C17	1.467 (3)	C11—C12	1.378 (4)
C4—C5	1.341 (3)	C11—H11	0.95
C4—O2	1.366 (3)	C24—C25	1.365 (5)
C17—C18	1.338 (3)	C24—H24	0.95
C17—O4	1.371 (3)	C12—C13	1.386 (4)
C5—C6	1.421 (4)	C12—H12	0.95
C5—H5	0.95	C25—C26	1.371 (4)
C18—C19	1.416 (4)	C25—H25	0.95
C18—H18	0.95	C13—H13	0.95
C6—C7	1.326 (3)	C26—H26	0.95
C6—H6	0.95	N2—H2	0.88
C19—C20	1.333 (4)	N4—H4	0.88
			
N1—C1—H1A	109.5	C9—C8—N1	119.7 (2)
N1—C1—H1B	109.5	C13—C8—N1	119.6 (2)
H1A—C1—H1B	109.5	C26—C21—C22	120.5 (3)
N1—C1—H1C	109.5	C26—C21—N3	119.4 (2)
H1A—C1—H1C	109.5	C22—C21—N3	119.9 (2)
H1B—C1—H1C	109.5	C8—C9—C10	119.6 (2)
N3—C14—H14A	109.5	C8—C9—H9	120.2
N3—C14—H14B	109.5	C10—C9—H9	120.2
H14A—C14—H14B	109.5	C21—C22—C23	118.3 (3)
N3—C14—H14C	109.5	C21—C22—H22	120.8
H14A—C14—H14C	109.5	C23—C22—H22	120.8
H14B—C14—H14C	109.5	C9—C10—C11	120.1 (3)
N1—C2—N2	114.7 (2)	C9—C10—H10	119.9
N1—C2—S1	125.7 (2)	C11—C10—H10	119.9
N2—C2—S1	119.6 (2)	C24—C23—C22	120.3 (3)
N3—C15—N4	116.2 (2)	C24—C23—H23	119.8
N3—C15—S2	125.0 (2)	C22—C23—H23	119.8
N4—C15—S2	118.77 (19)	C12—C11—C10	120.0 (3)
O1—C3—N2	121.7 (2)	C12—C11—H11	120
O1—C3—C4	124.1 (2)	C10—C11—H11	120
N2—C3—C4	114.2 (2)	C25—C24—C23	119.9 (3)
O3—C16—N4	123.3 (2)	C25—C24—H24	120
O3—C16—C17	123.5 (2)	C23—C24—H24	120
N4—C16—C17	113.1 (2)	C11—C12—C13	120.3 (3)
C5—C4—O2	109.6 (2)	C11—C12—H12	119.9
C5—C4—C3	134.1 (2)	C13—C12—H12	119.9
O2—C4—C3	116.2 (2)	C24—C25—C26	120.7 (3)
C18—C17—O4	109.9 (2)	C24—C25—H25	119.7
C18—C17—C16	133.2 (2)	C26—C25—H25	119.7
O4—C17—C16	116.7 (2)	C12—C13—C8	119.3 (3)
C4—C5—C6	107.1 (2)	C12—C13—H13	120.4
C4—C5—H5	126.4	C8—C13—H13	120.4
C6—C5—H5	126.4	C25—C26—C21	120.2 (3)
C17—C18—C19	107.2 (2)	C25—C26—H26	119.9
C17—C18—H18	126.4	C21—C26—H26	119.9
C19—C18—H18	126.4	C7—O2—C4	106.06 (19)
C7—C6—C5	105.8 (2)	C20—O4—C17	105.79 (19)
C7—C6—H6	127.1	C2—N1—C8	122.3 (2)
C5—C6—H6	127.1	C2—N1—C1	120.6 (2)
C20—C19—C18	106.3 (2)	C8—N1—C1	116.8 (2)
C20—C19—H19	126.8	C15—N3—C21	124.2 (2)
C18—C19—H19	126.8	C15—N3—C14	118.7 (2)
C6—C7—O2	111.4 (2)	C21—N3—C14	116.0 (2)
C6—C7—H7	124.3	C3—N2—C2	122.2 (2)
O2—C7—H7	124.3	C3—N2—H2	118.9
C19—C20—O4	110.8 (2)	C2—N2—H2	118.9
C19—C20—H20	124.6	C16—N4—C15	123.24 (19)
O4—C20—H20	124.6	C16—N4—H4	118.4
C9—C8—C13	120.6 (3)	C15—N4—H4	118.4
			
O1—C3—C4—C5	156.2 (3)	N3—C21—C26—C25	−174.3 (2)
N2—C3—C4—C5	−23.3 (4)	C6—C7—O2—C4	−0.3 (3)
O1—C3—C4—O2	−20.7 (4)	C5—C4—O2—C7	−0.3 (3)
N2—C3—C4—O2	159.7 (2)	C3—C4—O2—C7	177.3 (2)
O3—C16—C17—C18	165.5 (3)	C19—C20—O4—C17	0.0 (3)
N4—C16—C17—C18	−11.8 (4)	C18—C17—O4—C20	0.4 (3)
O3—C16—C17—O4	−9.2 (4)	C16—C17—O4—C20	176.3 (2)
N4—C16—C17—O4	173.5 (2)	N2—C2—N1—C8	−14.4 (3)
O2—C4—C5—C6	0.8 (3)	S1—C2—N1—C8	166.46 (18)
C3—C4—C5—C6	−176.3 (3)	N2—C2—N1—C1	170.9 (2)
O4—C17—C18—C19	−0.7 (3)	S1—C2—N1—C1	−8.2 (3)
C16—C17—C18—C19	−175.7 (3)	C9—C8—N1—C2	126.6 (3)
C4—C5—C6—C7	−0.9 (3)	C13—C8—N1—C2	−56.9 (3)
C17—C18—C19—C20	0.7 (3)	C9—C8—N1—C1	−58.6 (3)
C5—C6—C7—O2	0.8 (3)	C13—C8—N1—C1	118.0 (3)
C18—C19—C20—O4	−0.4 (3)	N4—C15—N3—C21	19.3 (3)
C13—C8—C9—C10	1.4 (4)	S2—C15—N3—C21	−161.15 (19)
N1—C8—C9—C10	177.9 (2)	N4—C15—N3—C14	−173.3 (2)
C26—C21—C22—C23	−0.2 (4)	S2—C15—N3—C14	6.2 (3)
N3—C21—C22—C23	175.1 (2)	C26—C21—N3—C15	−129.9 (3)
C8—C9—C10—C11	−0.3 (4)	C22—C21—N3—C15	54.7 (3)
C21—C22—C23—C24	−0.7 (4)	C26—C21—N3—C14	62.4 (3)
C9—C10—C11—C12	−0.2 (4)	C22—C21—N3—C14	−112.9 (3)
C22—C23—C24—C25	0.7 (5)	O1—C3—N2—C2	0.3 (4)
C10—C11—C12—C13	−0.4 (4)	C4—C3—N2—C2	179.9 (2)
C23—C24—C25—C26	0.1 (5)	N1—C2—N2—C3	−66.0 (3)
C11—C12—C13—C8	1.4 (4)	S1—C2—N2—C3	113.1 (2)
C9—C8—C13—C12	−2.0 (4)	O3—C16—N4—C15	−21.2 (4)
N1—C8—C13—C12	−178.5 (2)	C17—C16—N4—C15	156.1 (2)
C24—C25—C26—C21	−1.0 (4)	N3—C15—N4—C16	61.9 (3)
C22—C21—C26—C25	1.0 (4)	S2—C15—N4—C16	−117.7 (2)

### Hydrogen-bond geometry (Å, °)

**Table:**

*D*—H···*A*	*D*—H	H···*A*	*D*···*A*	*D*—H···*A*
N2—H2···O3^i^	0.88	2.54	3.331 (3)	149
N4—H4···O1^ii^	0.88	2.17	3.010 (2)	159
C5—H5···O3^i^	0.95	2.43	3.341 (3)	161
C5—H5···O4^i^	0.95	2.46	3.137 (3)	128
C14—H14C···O2	0.98	2.59	3.227 (4)	123
C18—H18···O1^ii^	0.95	2.44	3.274 (3)	147
C18—H18···O2^ii^	0.95	2.55	3.167 (3)	123

Symmetry codes: (i) −*x*, *y*+1/2, −*z*+1/2; (ii) −*x*+1, *y*−1/2, −*z*+1/2.

## References

[bb1] Estévez-Hernández, O., Hidalgo, J. L., Reguera, E. & Naranjo, I. (2007). *Sens. Actuators B*, **120**, 766–772.

[bb2] Farrugia, L. J. (1997). *J. Appl. Cryst.* **30**, 565.

[bb3] Farrugia, L. J. (1999). *J. Appl. Cryst.* **32**, 837–838.

[bb4] Koch, K. R., Sacht, C. & Bourne, S. (1995). *Inorg. Chim. Acta*, **232**, 109–115.

[bb5] Morales, A. D., García-Granda, S., Esteva, J. R., Stevens, A. P. & Crespo, G. A. A. (1997). *Acta Cryst.* C**53**, IUC9700019.

[bb6] Nonius (2000). *COLLECT* Nonius BV, Delft, The Netherlands.

[bb7] Otazo, E., Pérez, L., Estévez, O., Rojas, S. & Alonso, J. (2001). *J. Chem. Soc. Perkin Trans. 2*, pp. 2211–2218.

[bb8] Otwinowski, Z. & Minor, W. (1997). *Methods in Enzymology*, Vol. 276, *Macromolecular Crystallography*, Part A, edited by C. W. Carter Jr & R. M. Sweet, pp. 307–326. New York: Academic Press.

[bb9] Sheldrick, G. M. (2008). *Acta Cryst* A**64**, 112–122. 10.1107/S0108767307043930 18156677

